# Supplementary material for: Physical Chemistry of Chloroquine Permeation through the Cell Membrane with Atomistic Detail
Source: J Chem Inf Model. 2023 Nov 10;63(22):7124–32. doi: 10.1021/acs.jcim.3c01363 (PMC10685453; doi:10.1021/acs.jcim.3c01363)
Supplement: Supplementary file 1 — ci3c01363_si_001.pdf [file ci3c01363_si_001.pdf]

Supporting Information: Physical Chemistry of Chloroquine Permeation through the Cell Membrane with Atomistic Detail

Mirko Paulikat,[†] GiovanniMaria Piccini,[‡] Emiliano Ippoliti,[†] Giulia Rossetti,[†,¶,§] Fabio Arnesano,[∥] and Paolo Carloni[*,†,⊥]

†*Computational Biomedicine, Institute of Advanced Simulations IAS-5/Institute for Neuroscience and Medicine INM-9, Forschungszentrum Jülich GmbH, 52428 Jülich, Germany*

‡*Institute of Technical and Macromolecular Chemistry, RWTH Aachen University, 52074 Aachen, Germany*

¶*Jülich Supercomputing Centre (JSC), Forschungszentrum Jülich GmbH, 52428 Jülich, Germany*

§*Department of Neurology, RWTH Aachen University, 52062 Aachen, Germany*

∥*Department of Chemistry, University of Bari "Aldo Moro", 70125 Bari, Italy*

⊥*Department of Physics, RWTH Aachen University, 52062 Aachen, Germany*

E-mail: p.carloni@fz-juelich.de

S1. MD Simulations of M1 and M2

The pure POPC (**M1**) and mixed POPC/POPS (**M2**) bilayers were subject to 60 and 110 ns MD simulations (at 310 K and 1 bar), respectively. The potential energies, temperatures and box dimensions are shown in Figures S1 and S2 for **M1** and **M2**, respectively. All quantities relax within the first nanoseconds. Furthermore, the averaged mass densities of the systems and the corresponding contributions from water molecules, lipid headgroups and tails are calculated from the last 50 ns of the simulations along the membrane normal (Figures S1d and S2d). As expected, the highest mass densities are found in the headgroup regions at around ±20 Å. Water molecules cannot be detected in the hydrophobic cores of both lipid bilayers (between −10 and 10 Å).

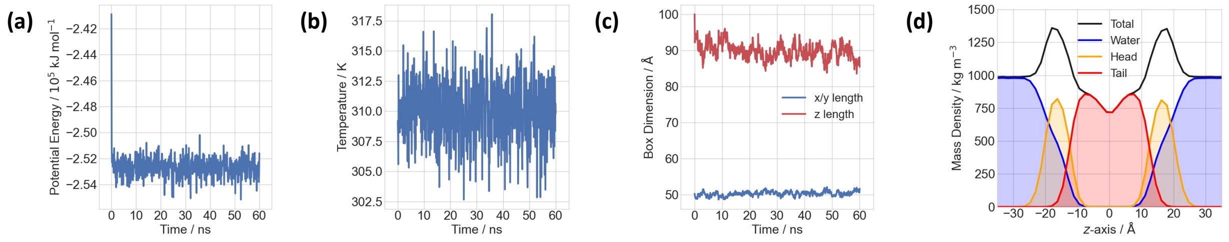

Figure S1: Some features of the 60 ns NPT MD simulation of the POPC bilayer (**M1**). The (a) potential energy, (b) the temperature and (c) the box dimensions of the x/y-axes (blue) and z-axis (red) are monitored with the simulation time. (d) Averaged mass density from the last 50 ns of the MD simulation projected onto the membrane normal (z-axis). The total mass density (black) and the contributions from water molecules (blue), POPC headgroups (orange) and POPC tails (red) are shown.

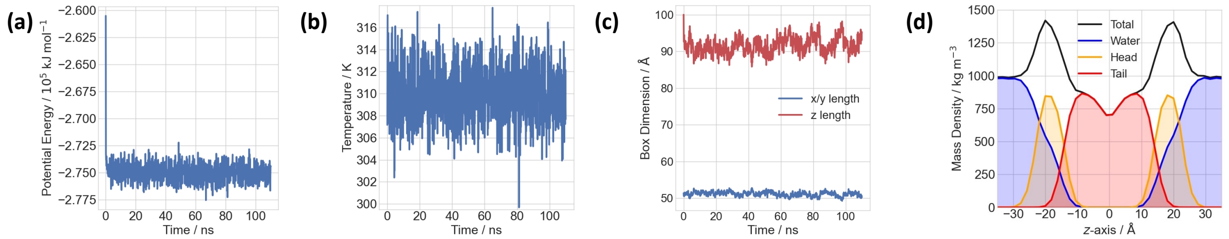

Figure S2: Same features as those for **M1** (Figure S1) for the 110 ns NPT MD simulation of the POPC/POPS mixed bilayer (**M2**). The plots report the same quantities with the same color coding. In (d), the contributions from the POPC/POPS headgroups (orange) and POPC/POPS tails (red) are shown.

S2. CQ^0 Permeation through a POPC Membrane

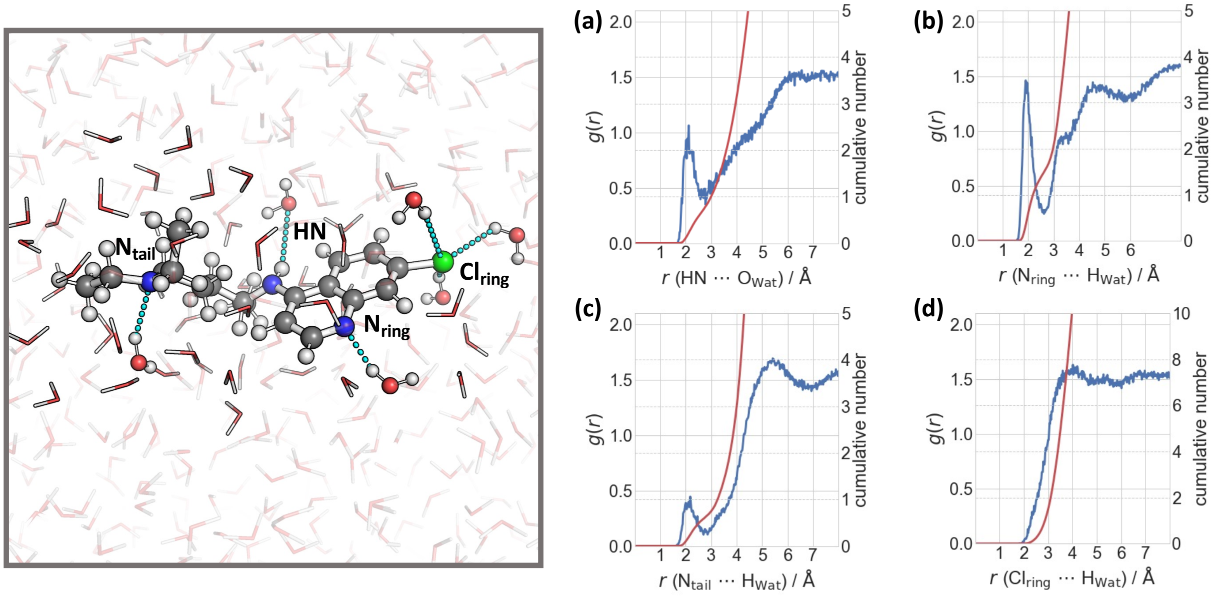

Figure S3: **CQ^0** in the solvent state. Left: Representative snapshot of **CQ^0** in the aqueous phase, labeling its H-bond acceptor and donor atoms. Dashed lines indicate H-bond interactions with water molecules. Right: Radial distribution functions and coordination numbers of H-bond interactions for (a) $\mathrm{HN}\cdots\mathrm{O_{Wat}}$, (b) $\mathrm{N_{ring}}\cdots\mathrm{H_{Wat}}$, (c) $\mathrm{N_{tail}}\cdots\mathrm{H_{Wat}}$, and (d) $\mathrm{Cl_{ring}}\cdots\mathrm{H_{Wat}}$ of **CQ^0** in the aqueous phase.

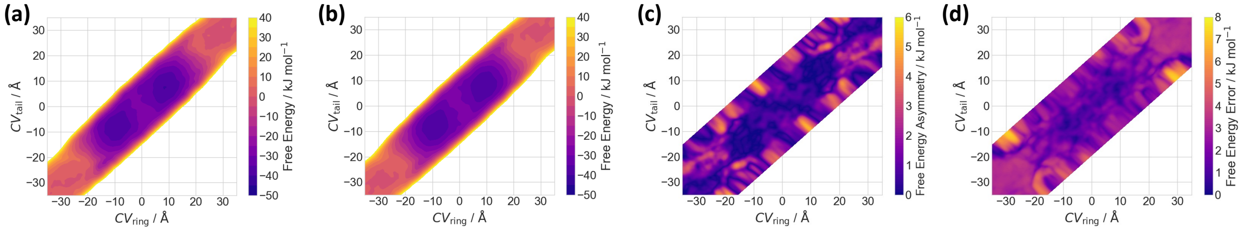

Figure S4: Analysis of the free energy surface for the permeation process of **CQ^0** through the POPC membrane. (a) Free energy surface as a function of $\mathrm{CV_{ring}}$ and $\mathrm{CV_{tail}}$ (see Figure 1 in the main text). (b) Symmetrized free energy surface: $G_{\mathrm{sym}} = 1/2[G(\mathrm{CV_{ring}}, \mathrm{CV_{tail}}) + G(-\mathrm{CV_{ring}}, -\mathrm{CV_{tail}})]$. (c) Asymmetry analysis of the free energy surface: $G_{\mathrm{asym}} = \mathrm{abs}[G(\mathrm{CV_{ring}}, \mathrm{CV_{tail}}) - G(-\mathrm{CV_{ring}}, -\mathrm{CV_{tail}})]$. (d) Estimated free energy error from block averaging of the symmetrized free energy profile.

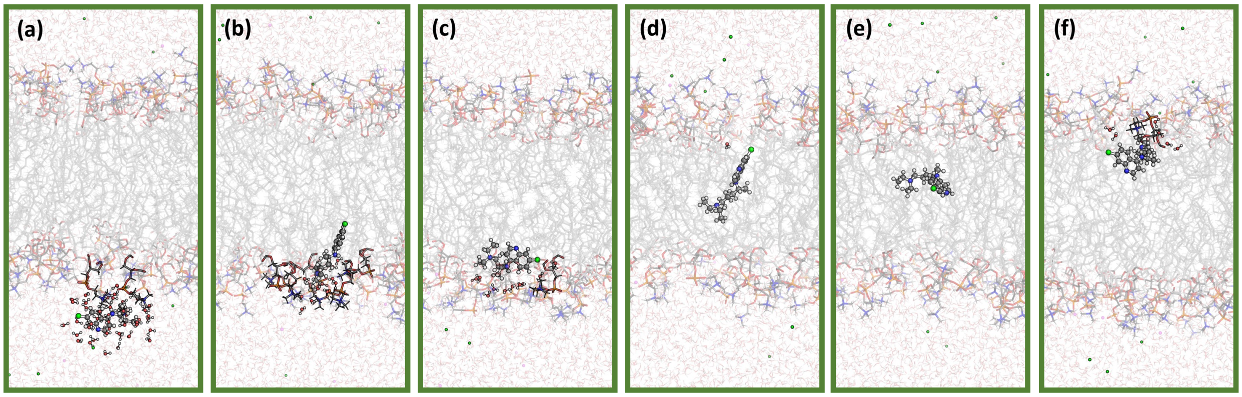

Figure S5: (a)–(f) Representative snapshots of the $\mathbf{CQ^0}$ permeation process across the POPC membrane.

S3. CQ^+ Permeation through a POPC Membrane

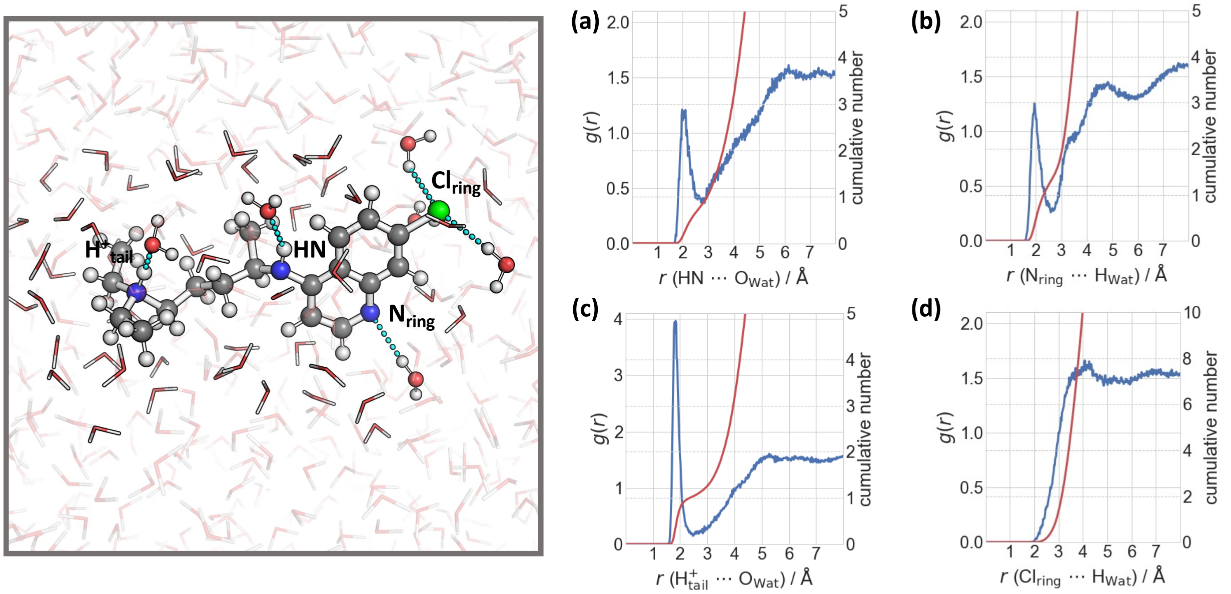

Figure S6: $\mathbf{CQ^+}$ in the solvent state. Left: Representative snapshot of $\mathbf{CQ^+}$ in the aqueous phase, labeling its H-bond acceptor and donor atoms. Dashed lines indicate H-bond interactions with water molecules. Right: Radial distribution functions and coordination numbers of H-bond interactions for (a) $\mathrm{HN}\cdots\mathrm{O_{Wat}}$, (b) $\mathrm{N_{ring}}\cdots\mathrm{H_{Wat}}$, (c) $\mathrm{H^{+}{}_{tail}}\cdots\mathrm{O_{Wat}}$, and (d) $\mathrm{Cl_{ring}}\cdots\mathrm{H_{Wat}}$.

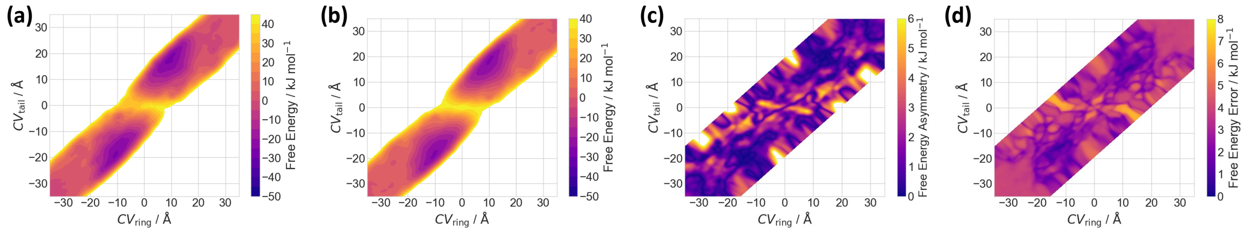

Figure S7: Analysis of the free energy surface for the permeation process of **CQ**$^+$ through the POPC membrane. (a) Free energy surface as a function of $\mathrm{CV_{ring}}$ and $\mathrm{CV_{tail}}$ (see Figure 1 in the main text). (b) Symmetrized free energy surface: $G_{\mathrm{sym}} = 1/2[G(\mathrm{CV_{ring}}, \mathrm{CV_{tail}}) + G(-\mathrm{CV_{ring}}, -\mathrm{CV_{tail}})]$. (c) Asymmetry analysis of the free energy surface: $G_{\mathrm{asym}} = \mathrm{abs}[G(\mathrm{CV_{ring}}, \mathrm{CV_{tail}}) - G(-\mathrm{CV_{ring}}, -\mathrm{CV_{tail}})]$. (d) Estimated free energy error from block averaging of the symmetrized free energy profile.

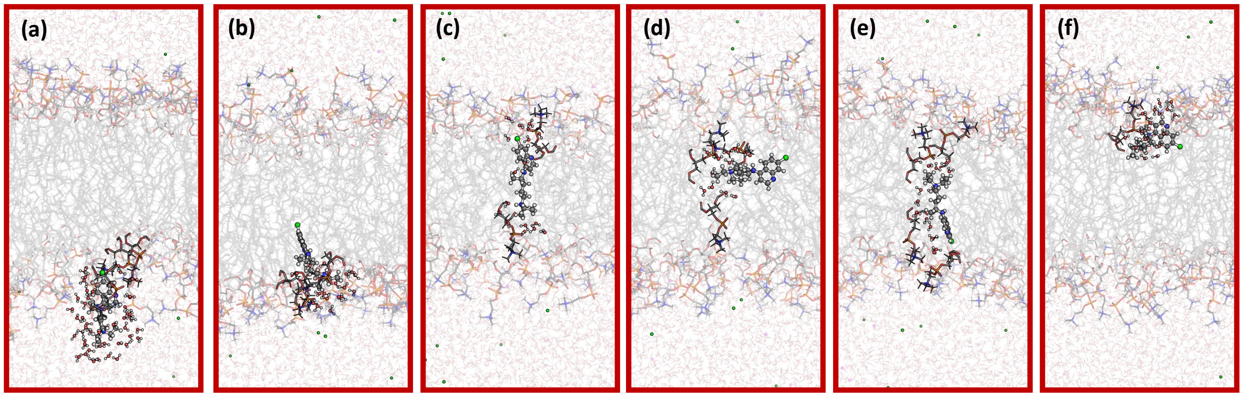

Figure S8: (a)–(f) Representative snapshots of the **CQ**$^+$ permeation process across the POPC membrane.

S4. $\mathbf{CQ^0}\rightleftharpoons\mathbf{CQ^+}$ Permeation through a POPC Membrane

We used the thermodynamic cycle depicted in Figure S9a to calculate the free energy for the proton transfer, and its associated pK_a value, in the lipid phase.[S1] The transfer free energies from the aqueous to the lipid phase for $\mathbf{CQ^0}$ ($\Delta G_\mathrm{trans;neutral}$) and $\mathbf{CQ^+}$ ($\Delta G_\mathrm{trans;charged}$) were calculated by the well-tempered metadynamics simulations. The term $\Delta G_{\mathbf{W};\mathrm{H^+-transfer}}$ corresponds to the deprotonation free energy of $\mathbf{CQ^+}$ in the aqueous phase, which is calculated by

$$\Delta G_{\mathbf{W};\mathrm{H^+-transfer}} = \ln(10)RT(\mathrm{p}K_\mathrm{a} - \mathrm{pH}) \quad . \tag{1}$$

Here, R and T are the universal gas constant and temperature, respectively. The experimental pK_a of 10.4[S2] and a neutral pH (7) were used. The free energy difference between $\mathbf{CQ^+}$ and $\mathbf{CQ^0}$ then amounts to 20.2 kJ mol^{-1} at 310 K. This value was used as a constant shift for the calculated free energy surface of $\mathbf{CQ^0}$.

Summing up the individual terms, the deprotonation free energy of $\mathbf{CQ^+}$ could be determined for the lipid phase ($\Delta G_{\mathbf{L};\mathrm{H^+-transfer}}$). The associated p$K_\mathrm{a}$ value is then given by

$$\mathrm{p}K_\mathrm{a} = \frac{\Delta G_{\mathbf{L};\mathrm{H^+-transfer}}}{\ln(10)RT} + \mathrm{pH} \quad . \tag{2}$$

Finally, the fraction of $\mathbf{CQ^+}$ in the different environments is given by

$$\frac{[\mathbf{CQ^+}]}{[\mathbf{CQ^0}] + [\mathbf{CQ^+}]} = \frac{\exp(-\beta G_{\mathbf{L};\mathrm{H^+-transfer}})}{1 + \exp(-\beta G_{\mathbf{L};\mathrm{H^+-transfer}})} \tag{3}$$

where β is the reciprocal thermodynamic temperature $(RT)^{-1}$. This fraction of $\mathbf{CQ^+}$, and its complementary one for $\mathbf{CQ^0}$, were used to weight the free energy surfaces of $\mathbf{CQ^+}$ and $\mathbf{CQ^0}$ to determine the protonation-dependent $\mathbf{CQ^0} \rightleftharpoons \mathbf{CQ^+}$ free energy surface.

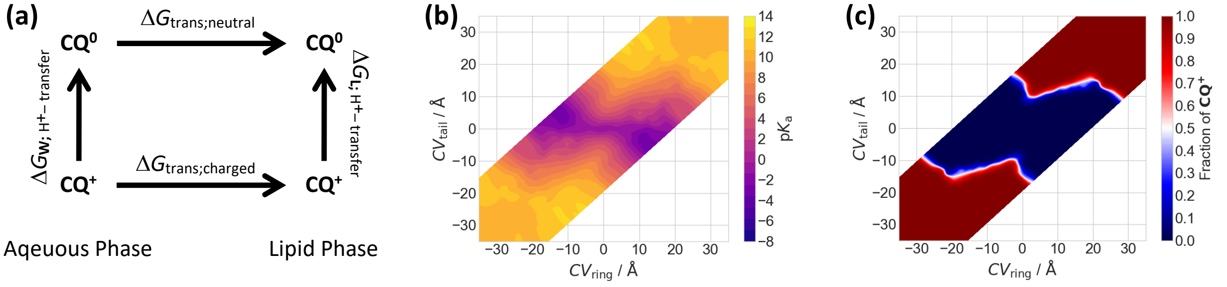

Figure S9: Analysis of the position-dependent equilibrium of $\mathbf{CQ^0}$ and $\mathbf{CQ^+}$ for the permeation through the POPC membrane. (a) Thermodynamic cycle for the calculation of the free energy difference $\Delta G_{\mathbf{L};\mathrm{H}^+-\mathrm{transfer}}$ of $\mathbf{CQ^0}$ and $\mathbf{CQ^+}$ in the lipid phase. The deprotonation free energy $\Delta G_{\mathbf{W};\mathrm{H}^+-\mathrm{transfer}}$ in the aqueous phase was calculated from Equation (1), while the two $\Delta G_{\mathrm{trans}}$ were calculated from well-tempered metadynamics simulations (see text above). (b) pK_a value of **CQ** as a function of $\mathrm{CV_{ring}}$ and $\mathrm{CV_{tail}}$ (see Figure 1 in the main text), calculated from Equation (2). (c) Fraction of $\mathbf{CQ^+}$ as a function of $\mathrm{CV_{ring}}$ and $\mathrm{CV_{tail}}$, calculated from Equation (3).

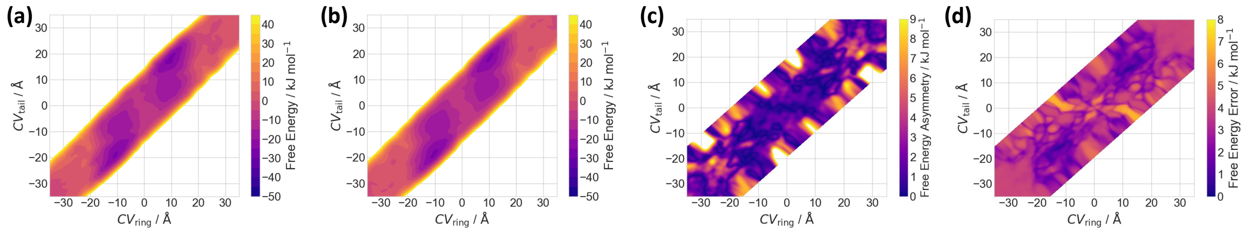

Figure S10: Analysis of the free energy surface for the permeation process of $\mathbf{CQ^0} \rightleftharpoons \mathbf{CQ^+}$ through the POPC membrane. (a) Free energy surface as a function of $\mathrm{CV_{ring}}$ and $\mathrm{CV_{tail}}$ (see Figure 1 in the main text). (b) Symmetrized free energy surface: $G_{\mathrm{sym}} = 1/2[G(\mathrm{CV_{ring}}, \mathrm{CV_{tail}}) + G(-\mathrm{CV_{ring}}, -\mathrm{CV_{tail}})]$. (c) Asymmetry analysis of the free energy surface: $G_{\mathrm{asym}} = \mathrm{abs}[G(\mathrm{CV_{ring}}, \mathrm{CV_{tail}}) - G(-\mathrm{CV_{ring}}, -\mathrm{CV_{tail}})]$. (d) Estimated free energy error from block averaging of the symmetrized free energy profile.

S5. CQ^0 Permeation through a Mixed POPC/POPS Membrane

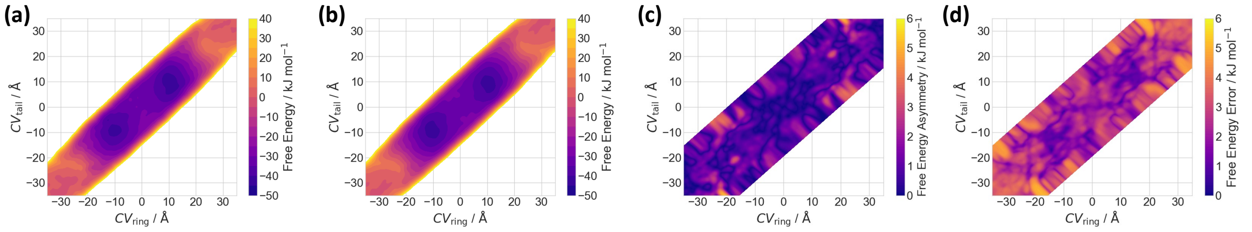

Figure S11: Analysis of the free energy surface for the permeation process of $\mathbf{CQ^0}$ through the POPC/POPS membrane. (a) Free energy surface as a function of $\mathrm{CV_{ring}}$ and $\mathrm{CV_{tail}}$ (see Figure 1 in the main text). (b) Symmetrized free energy surface: $G_{\mathrm{sym}} = 1/2[G(\mathrm{CV_{ring}}, \mathrm{CV_{tail}}) + G(-\mathrm{CV_{ring}}, -\mathrm{CV_{tail}})]$. (c) Asymmetry analysis of the free energy surface: $G_{\mathrm{asym}} = \mathrm{abs}[G(\mathrm{CV_{ring}}, \mathrm{CV_{tail}}) - G(-\mathrm{CV_{ring}}, -\mathrm{CV_{tail}})]$. (d) Estimated free energy error from block averaging of the symmetrized free energy profile.

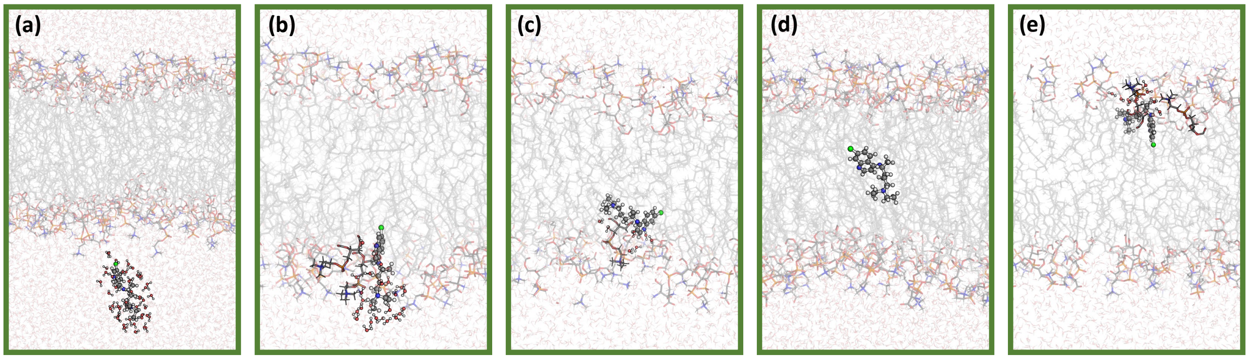

Figure S12: (a)–(f) Representative snapshots of the $\mathbf{CQ^0}$ permeation process across the POPC/POPS membrane.

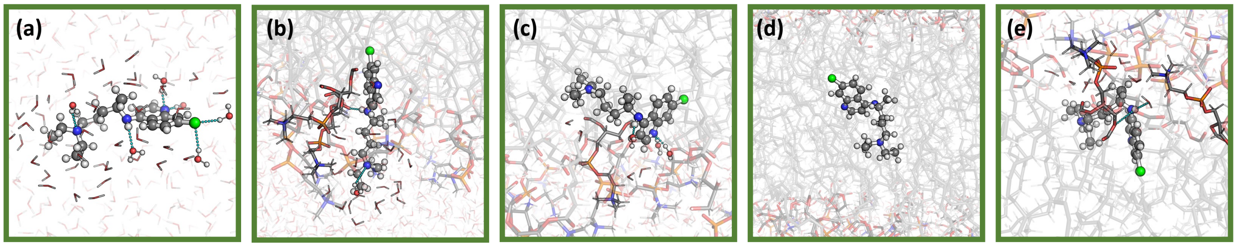

Figure S13: (a)–(e): Representative snapshots of the $\mathbf{CQ^0}$ permeation process across the POPC/POPS membrane, showing the interactions of $\mathbf{CQ^0}$ with water molecules and POPC/POPS headgroups.

S6. CQ^0 Permeation through a POPC Membrane with an External Field

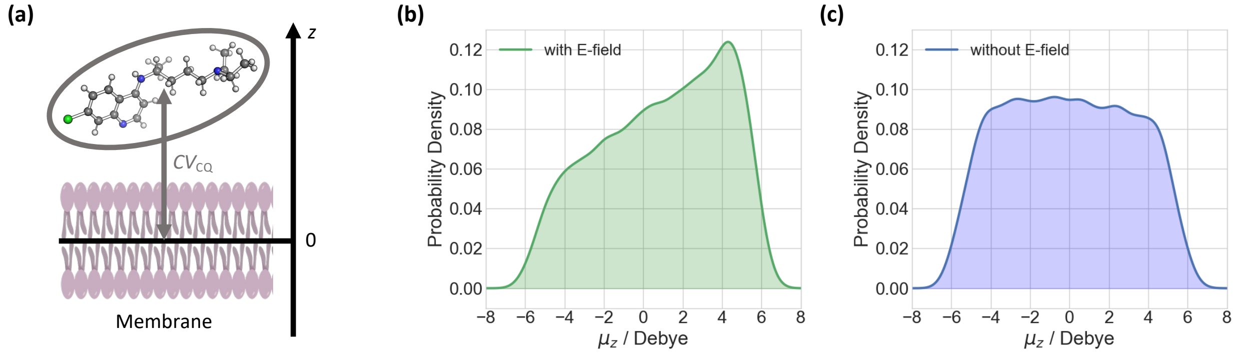

Figure S14: (a) Schematic representation of the employed CV to determine the impact of an external electric field. (b) Distribution of the z-component of the dipole moment (μ_Z) of $\mathbf{CQ^0}$ from well-tempered metadynamics simulations with an external electric field of 10 mV Å^{-1}. (c) Distribution of the z-component of the dipole moment (μ_Z) of $\mathbf{CQ^0}$ from well-tempered metadynamics simulations without an external electric field.

S7. Calculation of Properties

Diffusion Coefficients. The position-dependent diffusion coefficients $D(z_i)$ of the drugs can be derived from the solution of the generalized Langevin equation (GLE), in which the drug is considered as a harmonic oscillator in a frictional bath.[S3,S4] The diffusion coefficient of the drug reads:[S3,S4]

$$D(z_i) = \lim_{s \to 0} \frac{-\mathcal{L}\{C_v(t)\}(s) \cdot \langle \delta z^2 \rangle_i \langle \dot{z}^2 \rangle_i}{\mathcal{L}\{C_v(t)\}(s) \cdot [s\langle \delta z^2 \rangle_i + s^{-1} \langle \dot{z}^2 \rangle_i] - \langle \delta z^2 \rangle_i \langle \dot{z}^2 \rangle_i} \quad (4)$$

where z corresponds to the distance of the molecule to the membrane center, as defined by the collective variable (see Figure 1 in the main text). δz is the displacement from its average position, $\langle z^2 \rangle$ and $\langle \dot{z}^2 \rangle$ are the variances of the position and the velocity, respectively. The

velocity autocorrelation function $C_v(t)$ is numerically calculated as

$$C_v(t) = \langle \dot{z}(0)\dot{z}(t) \rangle = \frac{1}{n}\sum_{j=0}^{n} \dot{z}(j \cdot \Delta t)\dot{z}(t + j \cdot \Delta t) \tag{5}$$

where n is the number of sampled points and Δt the time step. Its Laplace transform $\mathcal{L}\{C_v(t)\}(s)$ is given by

$$\mathcal{L}\{C_v(t)\}(s) = \int_0^\infty C_v(t) \exp(st) \mathrm{d}t \tag{6}$$

which is a function of the transformation parameter s.

Equation (4) was solved numerically following the protocol in Ref. S5. The drugs were restrained using a harmonic potential along the z-axis (see Figure S14a). The drugs were then pulled through the membrane to the opposite site (at +40 Å) using a moving harmonic restraint with a force constant of 100 kJ mol^{-1} Å^{-2} in 80 ns *NPT* simulations. The procedure was carried out for 3 replicas for $\mathbf{CQ^0}$ and $\mathbf{CQ^+}$, considering the POPC membrane, as well as for $\mathbf{CQ^0}$ across the mixed POPC/POPS membrane.

In a first set of calculations, we determined the full diffusion coefficient profile of $\mathbf{CQ^0}$ across the POPC membrane. From the pulling simulations, snapshots were taken in 1 ns intervals. This leads to 81 windows in 1 Å steps in the range of z = [–40 Å; +40 Å]. For each window i, the system was re-equilibrated for 10 ns *NPT* simulation by restraining the position of $\mathbf{CQ^0}$ at z_i with a harmonic potential of force constant 125 kJ mol^{-1} Å^{-2}, following 5 ns production time in the *NVE* ensemble. The MD data of each of the 81 windows were numerically processed according to Equations (4–6) for s ranging from 0.005 to 0.1 fs^{-1}, following a procedure similar to that of Ref. S5. An almost linear segment was defined by a curvature of the diffusion coefficient (as a function of the parameter s) lower than 0.01 Å^2 fs. The position-dependent diffusion coefficient $D(z_i)$ was then determined by the intercept of a linear regression from this segment. The final diffusion profile was obtained by averaging over the replicas and smoothening with a moving average using five windows. The results

are shown in Figure S15.

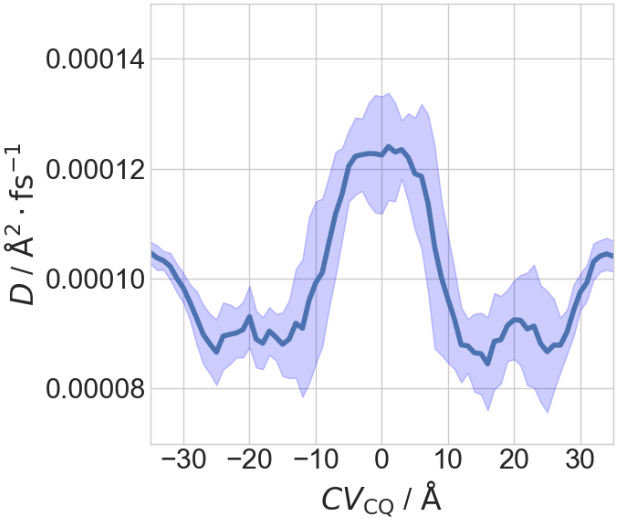

Figure S15: Position-dependent diffusion coefficient of CQ0 upon permeation through a POPC membrane.

In a second set of calculations, we determined the local diffusion coefficients along the minimum free energy path (MFEP) within our two dimensional CV space (see Figure 1 in the main text) for all systems. We followed the procedure described in Refs. S6,S7. Here, we only considered the positions at the free energy maxima, because these contribute most to the permeability of the drugs, as demonstrated in Refs. S6,S7, thereby significantly reducing the computational cost. We applied the same computational protocol as described above, except that a two dimensional harmonic potential was used to restrain the ring and tail moieties of the drugs (CV_{ring} and CV_{tail}, respectively). The deviations from the reference positions were projected onto the tangent of the MFEP to obtain a time series for the diffusion along permeation coordinate.[S6,S7] The latter was then processed to determine the diffusion coefficient, using the protocol as described above. The results are summarized in Table S1.

Table S1: Position-dependent diffusion coefficients of $\mathbf{CQ^0}$ and $\mathbf{CQ^+}$, considering the POPC and POPC/POPS membranes.

	System	CV_{ring} / Å	CV_{tail} / Å	D / 10^4 Å^2 fs^{-1}
$\mathbf{CQ^0}$	POPC	−22.69	−22.15	2.10 ± 0.16
$\mathbf{CQ^0}$	POPC/POPS	−22.59	−29.12	1.32 ± 0.02
$\mathbf{CQ^+}$	POPC	3.30	−0.73	1.63 ± 0.14
$\mathbf{CQ^+}$	POPC	−23.30	−24.80	1.51 ± 0.19

Permeability Coefficients. The permeability coefficient P was calculated from the inhomogeneous solubility-diffusion model by[S8]

$$\frac{1}{P} = \exp(-\beta G_{\text{ref}}) \int_{z_1}^{z_2} \frac{\exp(\beta G(z))}{D(z)} \text{d}z \tag{7}$$

where z corresponds to the permeation coordinate. $G(z)$ and $D(z)$ are the position-dependent free energy and diffusion coefficient, respectively, and β is the reciprocal thermodynamic temperature. G_{ref} is the free energy in the aqueous phase, which was appropriately shifted to be zero.

The integral was calculated along the minimum free energy path (MFEP), using the diffusion coefficients reported in Table S1. The integration boundaries $z_{1/2}$ correspond to the positions of **CQ** at $CV_{ring} = \pm 35$ Å and $CV_{tail} = \pm 35$ Å. For the simulation of $\mathbf{CQ^0}$ across the POPC membrane using the one-dimensional CV_{CQ}, the position-dependent diffusion profile was used (see Figure S14). The integration boundaries were set to $z_{1/2} = \pm 35$ Å. The integrations were done numerically, using the trapezoidal rule. The error was estimated by adding Gaussian distributed noise to the integrand, according to its mean value and standard deviation at each integration point. We integrated 10^7 sampled profiles to calculate the standard deviation of the permeability coefficient. The results are shown in Table S2.

Table S2: Permeability coefficients of **CQ**, considering the POPC and POPC/POPS membranes.

System			P / cm s^{-1}
$\mathbf{CQ^0}$	POPC	CV_{CQ}	27.6 ± 9.0
$\mathbf{CQ^0}$	POPC	(CV_{ring}, CV_{tail})	38.2 ± 7.8
$\mathbf{CQ^+}$	POPC	(CV_{ring}, CV_{tail})	$8.5\ 10^{-4} \pm 8.5\ 10^{-4}$
$\mathbf{CQ^0} \rightleftharpoons \mathbf{CQ^+}$	POPC	(CV_{ring}, CV_{tail})	26.0 ± 6.0
$\mathbf{CQ^0}$	POPC/POPS	(CV_{ring}, CV_{tail})	44 ± 12

References

(S1) MacCallum, J. L.; Bennett, W. F. D.; Tieleman, D. P. Distribution of Amino Acids in a Lipid Bilayer from Computer Simulations. *Biophys. J.* **2008**, *94*, 3393–3404.

(S2) Warhurst, D. C.; Steele, J. C.; Adagu, I. S.; Craig, J. C.; Cullander, C. Hydroxychloroquine is much less active than chloroquine against chloroquine-resistant *Plasmodium falciparum*, in agreement with its physicochemical properties. *J. Antimicrob. Chemother.* **2003**, *52*, 188–193.

(S3) Woolf, T. B.; Roux, B. Conformational Flexibility of o-Phosphorylcholine and o-Phosphorylethanolamine: A Molecular Dynamics Study of Solvation Effects. *J. Am. Chem. Soc.* **1994**, *116*, 5916–5926.

(S4) Schumaker, M. F.; Pomès, R.; Roux, B. A combined molecular dynamics and diffusion model of single proton conduction through gramicidin. *Biophys. J.* **2000**, *79*, 2840–2857.

(S5) Gaalswyk, K.; Awoonor-Williams, E.; Rowley, C. N. Generalized Langevin Methods for Calculating Transmembrane Diffusivity. *J. Chem. Theory Comput.* **2016**, *12*, 5609–5619.

(S6) Sun, R.; Han, Y.; Swanson, J. M.; Tan, J. S.; Rose, J. P.; Voth, G. A. Molecular trans-

port through membranes: Accurate permeability coefficients from multidimensional potentials of mean force and local diffusion constants. *J. Chem. Phys.* **2018**, *149*.

(S7) Shoji, A.; Kang, C.; Fujioka, K.; Rose, J. P.; Sun, R. Assessing the Intestinal Permeability of Small Molecule Drugs via Diffusion Motion on a Multidimensional Free Energy Surface. *J. Chem. Theory Comput.* **2022**, *18*, 503–515.

(S8) Venable, R. M.; Krämer, A.; Pastor, R. W. Molecular Dynamics Simulations of Membrane Permeability. *Chem. Rev.* **2019**, *119*, 5954–5997.
